# Characterization of flavored milk containing bitter orange peel extract and Gaz‐angubin

**DOI:** 10.1002/fsn3.1975

**Published:** 2020-10-29

**Authors:** Arghavan Jalilzadeh‐Afshari, Vajiheh Fadaei

**Affiliations:** ^1^ Department of Food Science & Technology Shahr‐e‐Qods Branch Islamic Azad University Tehran Iran

**Keywords:** antibacterial properties, antioxidant activity, bitter orange peel extract, flavored milk, Gaz‐angubin

## Abstract

In this study, the effects of adding Gaz‐angubin at three different levels (5%, 10%, and 15% w/v) and bitter orange peel extract with three different concentrations (0.025%, 0.050%, and 0.075% w/v) on selected characteristics of the flavored milk were investigated during 10‐day storage at 4°C. The results showed that increasing the level of Gaz‐angubin and bitter orange peel extract increased viscosity, antioxidant activity, and total polyphenol content, decreased total microbial count, and improved the sensory characteristics of the flavored milk (*p* < .05). Generally, the flavored milk sample containing 15% Gaz‐angubin and 0.075% bitter orange peel extract was selected as the best treatment.

## Graphical abstract

Gaz‐angubin could be considered as the potential antioxidant and antibacterial ingredient in healthy food products. The flavored milk containing 15% w/v Gaz‐angubin and 0.075% w/v bitter orange peel extract was selected as the best treatment.

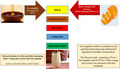

## 1 INTRODUCTION

Although consumption of milk and dairy products has been recommended as an important part of daily diet for people especially children, milk consumption has decreased especially among children. Most people prefer consumption of flavored milk, and the flavor type plays an important role in acceptability of flavored milk (De Pelsmaeker et al., 2013; Esmaeili & Ghani, 2017).

Gaz of Khunsar, the manna of Persia, is exuded by the last instar nymph of a small insect (*Cyamophila astragalicola Gegechkori*, Psyllidae) and collected from a spiny shrub (*Astragalus adscendens Boiss. & Haussk*., Leguminosae) which grows wild in western central lran (Grami, 1998; Wotton, 2010). The names Gaz and Gaz‐angubin have acquired generic status throughout Iran. Gaz of Khunsar, the sweet exudates with high fructose content, has been produced locally and consumed nationally in Iran for centuries. It is originally white or cream‐colored while may seem greenish or brownish yellow in bulk, depending on impurities. It is hydrophilic, soft, and very sticky under normal conditions, breakable when dry, and readily soluble in water and alcohol. Gaz‐angubin contains 41.2% fructose, 2% sucrose, 31.16% polysaccharide (which produces glucose, xylose, and mannose in response to acidic hydrolysis), 3.02% mucilage, gum, and 2.26% ash. Today, Gaz‐angubin could be considered as good sources of mineral essential elements (Yazdanparats et al., 2014) and one of the richest sources for fructose production; and as fructose is degraded without the need for insulin (by fructokinase enzyme), it is of great interest (Aynehchi, 1991). Gaz‐angubin is an anti‐tussive and treats chest congestion. It is also considered a diuretic and laxative (Ramezani et al., 2013; Samsam‐Shariat, 2004); and enjoys antioxidant activity (Siahpoosh et al., 2010).

The seed and peel of some fruits have a greater antioxidant activity in relation with their pulp (Guo et al., 2003). The different citrus peel by‐products are selectively enriched with high concentrations of different groups of flavonoid compounds (Garau et al., 2007; Manthey & Grohmann, 1996). Bitter orange (*Citrus aurantium L*) is a kind of citrus and extremely rich in flavonoids (Zeghad et al., 2019); its extract can be suitable for preventing Parkinson's disease in rats (Elyasi & Ghazvini, 2020). Bitter orange peel can be considered a suitable source of antioxidant containing large amounts of flavanone (Peterson et al., 2006). Chemical composition of bitter orange peel has been identified by Li et al. (2006).

As citrus peel extracts exhibit strong antioxidant activity, the use of these extracts in foods is recommended to suppress lipid oxidation. So far, various studies have been conducted on the relationship between antioxidant and antimicrobial effects of citrus peel extracts on inhibition of fat oxidation and prolongation of preservation of foods (Abd El‐aal & Halaweish, 2011; Adeline Dorcas et al., 2016; González‐Gómez et al., 2014; Hasani & Javadian, 2016; Mahmoud et al., 2016; Razmjoo et al., 2016; Rimini et al., 2014; Singh & Immanuel, 2014). However, no research has been reported on the effect of Gaz‐angubin in foods; Gaz of Khunsar and Gaz‐angubin has been used in confectionery in city of Ispahan [Isfahan] that specialized in making of a sweetmeat resembling nougat, also known as Gaz (Gaz is one of the most popular traditional sweets in Iran). Application of Gaz‐angubin in milk for production of flavored milk can cause improved dyspnea disorders and mitigate cough especially in children and the elderly, in metropolises, where dyspnea and pulmonary diseases are major problems for human health. The aim of this research was to investigate some physiochemical properties, overall acceptability, and total microbial count of a new flavored milk‐based beverage.

## 2 MATERIALS AND METHODS

### 2.1 Materials

Raw milk was prepared by Dairy workshop at Faculty of Agriculture, University of Tehran (Iran). Bitter orange peel was purchased from a local market in Mazandaran (Iran), and Gaz‐angubin was supplied by a local market in Khansar (Iran). Folin–Ciocalteu reagent, 2,2‐Di Phenyl‐1‐Picryl Hydrazyl (DPPH) reagent, and monohydrated Gallic acid were purchased from Sigma Co. (USA), and PC‐AGAR culture medium and other experimental chemicals used in this research were purchased from Merck Co., Germany.

### 2.2 Preparation of extract from bitter orange peel

First, according to Karsheva et al. (2013) method, 0.5 kg bitter orange peel was dried in an incubator in darkness for 4 days at 75°C and the samples were then milled. A total of 2.5 L hexane were mixed with bitter orange peel powder and exposed to 65°C for 4 hr in Bon Mari. Next, the mixture was passed through a filter paper. The remaining liquid was then placed inside a rotary at 45°C in order for hexane to evaporate. The residual substance was bitter orange peel extract.

### 2.3 Preparation of the flavored milk samples containing Gaz‐angubin and bitter orange peel

Raw milk with a fat content of 2.5% and solids‐non‐fat of 9.36% was supplied by Dairy Workshop in Faculty of Agriculture at University of Tehran. The milk was heated up to 50°C, and Gaz‐angubin as a sirup was added to the raw milk at three percentages (5%, 10%, 15% w/v). Next, weight‐volumetric solution of bitter orange peel extract was prepared with three dilutions (0.025%, 0.05%, and 0.075%) and added to milk containing Gaz‐angubin. Following mixing and homogenization of the mixture (40°C at 180 bar), the produced flavored milk samples were heated (75°C at 15 min) and packed in 250‐cc Pet dishes. The packaged samples of flavored milk were kept at 4°C after being cooled down.

The most important objective of the present research was to provide a functional product in such a way that it conclusively had the antioxidant and antimicrobial activities which are definitely found in Gaz‐angubin and bitter orange peel extract, that is, combining the Gaz‐angubin and bitter orange peel extract in the present research was on account of creating the pleasant odor and the sense of pleasure while drinking. Therefore, control samples were not considered in this study.

It is noteworthy that the chemical compositions of the bitter orange peel extract and Gaz‐angubin have been widely investigated by other researchers whose works were cited in the text of the present manuscript; so, we did not identify their chemical composition.

### 2.4 Flavored milk samples analysis

#### 2.4.1 Titratable acidity

Titratable acidity was determined as lactic acid by titrating with 0.1 N NaOH using phenolphthalein as an indicator (Gad et al., 2010).

#### 2.4.2 Viscosity

The viscosity of the samples was measured at 20°C using a Brookfield DV‐II + viscometer, and the spindle speed was set at 60 rpm. Readings were taken after the spindle had been rotating for 30 s, and the mean of three readings was recorded (Abdulghani et al., 2015).

#### 2.4.3 Total polyphenol content and antioxidant activity

To measure total polyphenol content (TPC), Folin‐Ciocalteu reagent method was used; in this experiment, absorbance of the samples was measured at the wavelength of 765 nm by a UV‐spectrophotometry device (model CARY 50, Australia; Elfalleh et al., 2012); TPC of the samples was calculated by standard curve of Gallic acid and stated as mg equivalent of Gallic acid per gram of the dried sample. The antioxidant activity of the flavored milk samples was evaluated using radical scavenging activity measurement method (RSA) with DPPH reagent and using spectrophotometry device (model CARY 50, Australia) at the wavelength of 517 nm (Elfalleh et al., 2012). The IC_50_ which denotes the amount (µl) of the plant extract in 1 ml solution required to reduce initial concentration of DPPH radicals by 50% was calculated. The results were expressed as IC_50_.

#### 2.4.4 Total microbial count

Total microbial count (TMC) was conducted using plate count agar by pour plate method according to Revelli et al. (2004). The dishes were incubated at 31°C for 48 hr in an incubator (ZN1434, Iran); and after that, the number of colonies was counted and expressed as log cfu/ml flavored milk.

#### 2.4.5 Sensory evaluations

Sensory evaluations were carried out to identify the best product. A total of 10 trained panelists (6 females and 4 males in the age range of 25–55 years) from Faculty of Agriculture, University of Tehran (Iran), were used in the analysis. The panelists were requested to evaluate the sensory properties according to a five‐point hedonic scale (Gamage et al., 2016). Since the final index assessment is overall acceptability; therefore, in this research, only overall acceptability results have been reported.

The flavored milk samples were taken for the analysis on 1, 5, and 10 days of storage.

### 2.5 Statistical analysis

Factorial experiment in a completely randomized block design (for overall acceptability) and completely randomized design (for physiochemical and microbial tests) was used. The experiment had three factors including antioxidant extract (at three levels of 0.025%, 0.05%, and 0.075% w/v), Gaz‐angubin (at three levels of 5%, 10%, and 15%), and time (1, 5, and 10 days). For each treatment, three replications were considered. In case of finding a significant difference between the treatments, least significant difference (Fisher's protected LSD) test at 0.05 level was used for comparing the means. Data analysis was performed by SAS 9.2 software.

## 3 RESULTS AND DISCUSSION

### 3.1 Titratable acidity of flavored milk samples during cold storage

According to results presented in Table 1, the greater the amount of Gaz‐angabin supplemented, the greater was acidity (*p* < .05); it can be related to the pH of Gaz‐angabin added. 1% water solution of Gaz‐angubin has a pH of 5.5 (Grami, 1998). Similar results to present study have been obtained by Kazemizadeh and Fadaei (2016) for flavored milk; they found that date sirup and pomegranate peel extract affect acidity of the final product. The high sugar compounds content in Gaz‐angabin (Aynehchi, 1991) and, as a result, the increase in lactic acid production due to increased access of microbial flora to the sugar source (Bensmira & Jiang, 2011; Cui et al., 2013; Dogan, 2011; Liu & Wen Lin, 2000; Milani & Koochaki, 2010) with increased concentration of Gaz‐angubin could be another reason for this.

**TABLE 1:** Titratable acidity (lactic acid%) of the flavored milk samples containing bitter orange peel extract and Gaz‐angubin during cold storage (mean ± *SD*)*

Day Treatment	0	5	10
C1G1	0.2025 ± 0.007^n^	0.1980 ± 0.008^p^	0.4860 ± 0.23^c^
C1G2	0.2070 ± 0.004^m^	0.2160 ± 0.019^i^	0.5217 ± 0.165^b^
C1G3	0.2070 ± 0.006^m^	0.2232 ± 0.016^g^	0.5850 ± 0.135^a^
C2G1	0.2025 ± 0.008^n^	0.2016 ± 0.010^o^	0.2167 ± 0.225^i^
C2G2	0.2078 ± 0.009^l^	0.2124 ± 0.016^j^	0.2243 ± 0.215^f^
C2G3	0.2115 ± 0.008^k^	0.2196 ± 0.015^h^	0.2793 ± 0.2^d^
C3G1	0.1926 ± 0.005^s^	0.1962 ± 0.009^r^	0.2160 ± 0.115^i^
C3G2	0.1933 ± 0.007^s^	0.1971 ± 0.013^q^	0.2250 ± 0.17^f^
C3G3	0.2025 ± 0.008^n^	0.2232 ± 0.014^f^	0.2340 ± 0.185^e^

Note

C = percentage of weight‐volumetric solution of bitter orange peel extract, G = Gaz‐angubin (%) (C1 = 0.025, C2 = 0.05; C3 = 0.075); (G1 = 5, G2 = 10; G3 = 15).

* Means with different subscripts differ significantly (*p* < .05).

An increase in the concentration of bitter orange peel extract induced a significant decrease in acidity (*p* < .05). It can be attributed to the antioxidant and antibacterial effects of the bitter orange peel extract (Adeline Dorcas et al., 2016; Hasani & Javadian, 2016) that prevent the activity of bacteria.

Over the storage time, the increment in acidity (*p* < .05) could attributed to acid lactic production by pasteurization‐resistant microorganisms. Furthermore, it can also be attributed to decreased phenolic compounds during storage time and, as a result, decreased antimicrobial properties of them (Al‐Rawahi et al., 2014; Ibrahium, 2010; Khan & Hanee, 2011; Rowayshed et al., 2013; Shiban et al., 2012). On the other hand, it seems that presence of Gaz‐angubin causes monosaccharide sugars to be provided for bacteria, thereby stimulating them to a slight extent and producing acid. Kazemizadeh and Fadaei (2016) also reported the decrease in pH of flavored milks containing date syrup and pomegranate peel extract during storage time with increased.

### 3.2 Viscosity of flavored milk samples during cold storage

The viscosity (Table 2) increased with increasing the percentage of Gaz‐angubin and bitter orange peel extract (*p* < .05). Gaz‐angubin contains large amounts of fructose sugar and polysaccharide (Aynehchi, 1991). Different polysaccharides with different structures (in terms of molecular weight, branching degree, and length of peripheral branches) influence the characteristics of rheology of drinks. Polysaccharides absorb water due to hydrophilic properties, causing diminished fluidity and increased viscosity of drinks. Addition of different concentrations of various hydrocolloids to drinks results in elevation of viscosity (Keshtkaran et al., 2012). Furthermore, with enhancement of Gaz‐angubin percentage, the acidity of flavored milk samples increased and on the other hand, with elevation of acidity, the viscosity also increased. With elevation of acidity, the probability of formation of casein network also increased, leading to further strength of the casein network and increased water absorptivity (Dogan, 2011; Liu & Wen Lin, 2000; Milani & Koochaki, 2010). With increased percentage of bitter orange peel extract, dry matter content of flavored milk samples increased (unreported data) and dry matter content and viscosity are directly related. These results confirm the results obtained by Boulenguer and Laurent (2003) and Koksoy and Kilic (2003) regarding the increase viscosity of acidic dairy drinks with elevation of dry matter content in them.

**TABLE 2:** Viscosity (cP) of the flavored milk samples containing bitter orange peel extract and Gaz‐angubin during cold storage (mean ± *SD*)*

Day Treatment	0	5	10
C1G1	3.22 ± 0.682^t^	2.97 ± 0.34^x^	2.87 ± 0.69^w^
C1G2	3.26 ± 0.462^s^	3.28 ± 0.833^r^	3.59 ± 0.495^m^
C1G3	3.61 ± 0.616^l^	3.72 ± 0.697^i^	3.80 ± 0.405^h^
C2G1	3.28 ± 0.858^r^	3.03 ± 0.442^z^	2.95 ± 0.675^y^
C2G2	3.66 ± 0.946^j^	3.64 ± 0.731^k^	3.51 ± 0.645^n^
C2G3	4.03 ± 0.88^d^	3.88 ± 0.68^g^	3.89 ± 0.6^f^
C3G1	3.47 ± 0.506^o^	3.30 ± 0.391^q^	3.05 ± 0.345^n^
C3G2	3.97 ± 0.748^e^	3.45 ± 0.578^p^	3.11 ± 0.51^u^
C3G3	5.36 ± 0.814^b^	4.65 ± 0.629^a^	4.04 ± 0.555^c^

Note

C = percentage of weight‐volumetric solution of bitter orange peel extract, G = Gaz‐angubin (%) (C1 = 0.025, C2 = 0.05; C3 = 0.075); (G1 = 5, G2 = 10; G3 = 15)

* Means with different subscripts differ significantly (*p* < .05).

There is a significant difference between the treatments during 10 days of storage with 5‐day intervals (*p* < .05). On the fifth and tenth days of storage, the viscosity of the treatment decreased in relation with postproduction time. This reduction was greater in the tenth day, which can be attributed to the increment of the product acidity (Panovska et al., 2012); reduction in pH causes contraction of the casein network (Senadeera et al., 2018).

### 3.3 Antioxidant activity and TPC of flavored milk samples during cold storage

The antioxidant activity (Table 3) and TPC (Table 4) of the different treatments have a significant difference (*p* < .05). With the increment of Gaz‐angubin and bitter orange peel extract percentages, the antioxidant activity and TPC of all the samples increased in each day of storage. Therefore, it can be concluded that Gaz‐angubin contains polyphenol compounds and has antioxidant activity (Siahpoosh et al., 2010). Moreover, the pulp and peel of bitter orange contain bioactive compounds such as flavonoids and terpenes with a high antioxidant activity (Adeline Dorcas et al., 2016; Garau et al., 2007; Nogata et al., 2006). Flavonoids belong to phenolic compounds (Thrugnanavel et al., 2007), which have important roles in health thanks to their pharmaceutical properties. These compounds have powerful antioxidant properties and inhibit free radicals and diminish the risk of some chronic diseases; further, they prevent some cardiovascular disorders (Gattuso et al., 2007; Parhiz et al., 2015). Also, their antiviral, antimicrobial, and anti‐allergic properties have also been proven (Cushine & Lamb, 2005). Antifungal effects of polymethoxylated flavones, isolated from the peels of *Citrus reticulata* Blanco, were identified (Wu et al., 2014). Gorinstein et al. (2006) stated that the antioxidant activity of citrus extracts is due to presence of carotenoids and ascorbic acid. Other researchers have identified flavonoid and phenolic antioxidant compounds in nonvolatile components of methanol extract of citrus peel (Guo et al., 2003). Nogata et al. (2006) reported that flavonoid compounds of bitter orange peel contain Naringin, Neoeriocitrin, Neohesperidin, Poncirin, Rhoifolin, and Neodiosmin. Bitter orange (*Citrus aurantium L*) is a kind of citrus, and its skin can be considered a suitable source of antioxidant containing large amounts of flavanone (Peterson et al., 2006). Flavanone glycosides, hesperidin, and naringin exist in large amounts, and some other flavonoids are present in small amounts in citrus fruits (Gorinstein et al., 2006). Thus, in each day of storage, the sample containing 15% Gaz‐angubin and 0.075% bitter orange peel extract (C3G3) has the maximum antioxidant activity, while the sample containing 5% Gaz‐angabin and 0.025% bitter orange peel extract (C1G1) has the lowest antioxidant activity.

**TABLE 3:** Antioxidant activity (µl/ml, IC_50_,) of the flavored milk samples containing bitter orange peel extract and Gaz‐angubin during Cold storage (mean ± *SD*)*

Day Treatment	0	5	10
C1G1	126.05 ± 17.204^l^	211.82 ± 27.553^e^	233.68 ± 9.401^a^
C1G2	99.16 ± 14.978^o^	113.09 ± 19.25^m^	224.27 ± 8.184^b^
C1G3	93.44 ± 15.18^p^	84.48 ± 28.08^r^	205.21 ± 8.295^g^
C2G1	89.62 ± 13.156^q^	166.81 ± 24.57^k^	222.15 ± 7.189^c^
C2G2	69.56 ± 16.698^u^	77.85 ± 28.958^s^	207.22 ± 9.125^f^
C2G3	55.36 ± 14.168 ^y^	59.59 ± 22.815^w^	191.52 ± 7.742^i^
C3G1	71.41 ± 16.192^t^	103.51 ± 26.325^n^	221.08 ± 8.848^c^
C3G2	41.41 ± 11.132^z^	66.24 ± 25.974^v^	201.40 ± 6.083^h^
C3G3	29.37 ± 15.888^A^	58.51 ± 29.835^x^	183.19 ± 8.682^j^

Note

C = percentage of weight‐volumetric solution of bitter orange peel extract, G = Gaz‐angubin (%) (C1 = 0.025, C2 = 0.05; C3 = 0.075); (G1 = 5, G2 = 10; G3 = 15).

* Means with different subscripts differ significantly (*p* < .05).

**TABLE 4:** TPC (mg GAE/g) of the flavored milk samples containing bitter orange peel extract and Gaz‐angubin during cold storage (mean ± *SD*)*

Day Treatment	0	5	10
C1G1	56.33 ± 10.761^jk^	44.67 ± 5.071^fghi^	32.33 ± 3.825^l^
C1G2	65.50 ± 9.368^hij^	53.66 ± 3.553^defg^	36.33 ± 3.33^kl^
C1G3	74.44 ± 9.495^cd^	65.67 ± 5.168^defg^	38.22 ± 3.375^kl^
C2G1	58.17 ± 8.229^ijk^	46.11 ± 4.522^efgh^	33.50 ± 2.925^l^
C2G2	68.11 ± 10.445^de^	66.67 ± 5.330^def^	38.33 ± 3.713^kl^
C2G3	82.17 ± 8.862^cd^	76.00 ± 4.199^bc^	39.78 ± 3.15^kl^
C3G1	84.11 ± 10.128^bc^	59.00 ± 4.845^efgh^	36.83 ± 3.6^kl^
C3G2	89.00 ± 6.963^ab^	67.83 ± 4.780^def^	44.56 ± 2.475 ^jk^
C3G3	98.11 ± 9.938^a^	83.17 ± 5.491^bc^	54.66 ± 3.533 ^ghij^

Note

C = percentage of weight‐volumetric solution of bitter orange peel extract, G = Gaz‐angubin (%) (C1 = 0.025, C2 = 0.05; C3 = 0.075); (G1 = 5, G2 = 10; G3 = 15).

* Means with different subscripts differ significantly (*p* < .05).

These results are in line with the findings obtained by other researchers regarding confirmation of antioxidant activity of citrus by‐products and existence of an inverse relationship between oxidation of foods and the extent of antioxidant activity of added citrus by‐products. For example, Hasani and Javadian (2016) concluded lowest oxidation content of lipid under the influence of high concentrations of bitter orange peel extract nanoencapsulated in carp fillets. Abd El‐aal and Halaweish (2011) also reported diminished rate of oxidation in soybean oil containing orange peel extract. Further, Rimini et al. (2014) confirmed antioxidant activity of thyme and orange essential oils blend and reported its effect of prevention from fat oxidation in chicken wing and chest meat. González‐Gómez et al. (2014) reported a significant decrease in apple juice by adding orange peel extract to it. The results of this research are in line with the results of Singh and Immanuel (2014) considering the effect of antioxidants of peel of pomegranate, lemon, and orange extracted on preventing oxidation of cheese fat through polyphenol compounds.

There is a significant decrease between antioxidant activity and TPC of treatments during cold storage (*p* < .05). Factors such as storage time and increasing temperature result in diminished antioxidant activity (Anese et al., 1999; Klimczak et al., 2007). Klimczak et al. (2007) indicated that over time, due to reduction in polyphenols and vitamin C, antioxidant activity also diminished. In accordance with these results, Mahmoud et al. (2016) reported diminished antioxidant activity of cakes containing lemon and orange peel extract nanoencapsulated during storage time. Also, this reduction could be contributed to increased interactions between milk proteins and polyphenols, thus lowering the number of free hydroxyls during storage time (Arts et al., 2002; Ozdal et al., 2013; Trigueros et al., 2014).

### 3.4 TMC of flavored milk samples during cold storage

Total microbial count of the different treatments (Table 5) has a significant difference (*p* < .05). In each day of storage, with increasing percentages of Gaz‐angubin and bitter orange peel extract, the TMC decreased in all the samples. Thus, it can be concluded that Gaz‐angubin is one of the Iranian mannas with antioxidant and antibacterial properties. Further, the external peel of bitter orange contains flavonoids (Nogata et al., 2006), which inhibit action of free radicals and are anti‐cancer, antioxidant, antiviral, antibacterial, and antifungal (Adeline Dorcas et al., 2016; Cushine & Lamb, 2005; Gattuso et al., 2007; Hasani & Javadian, 2016; Parhiz et al., 2015; Wu et al., 2014). The samples that have the maximum Gaz‐angubin percentage enjoy the greatest antimicrobial properties, as following: C3G3 (15% Gaz‐angubin and 0.075% bitter orange peel extract), C2G3 (15 Gaz‐angubin and 0.05% bitter orange peel extract), and C1G3 (15% Gaz‐angubin and 0.025% bitter orange peel extract). This indicates that with elevation of percentage of bitter orange peel extract, its antimicrobial properties also increase. The results of this research are congruent with the results obtained by González‐Gómez et al. (2014) considering a significant decrease in *Escherichia coli* and Listeria in apple juice with addition of orange peel extract to it. Also, Lin et al. (2010) reported that essential oil extracted from peels of sweet orange fruits could effectively inactivate *V. parahaemolyticus*, *S. typhimurium,* and *E. coli* on the food contact surfaces.

**TABLE 5:** TMC (log cfu/ml) of the flavored milk samples containing bitter orange peel extract and Gaz‐angubin during cold storage (mean ± *SD*)*

Day Treatment	0	5	10
C1G1	1.45 ± 0.714^c^	5.27 ± 6.861^c^	6.30 ± 9.231^a^
C1G2	1.43 ± 0.622^c^	4.77 ± 4.807^c^	6 ± 8.036^b^
C1G3	1.25 ± 0.63^c^	4.47 ± 6.992^c^	4.55 ± 8.145^c^
C2G1	1.43 ± 0.546^c^	5.23 ± 6.118^c^	6.21 ± 7.059^a^
C2G2	1.23 ± 0.693^c^	4.35 ± 7.211^c^	4.39 ± 8.960^c^
C2G3	1.04 ± 0.588^c^	3.18 ± 5.681^c^	3.53 ± 7.602^c^
C3G1	1.23 ± 0.672^c^	4.64 ± 6.555^c^	6.07 ± 8.688^b^
C3G2	1.04 ± 0.462^c^	3.92 ± 6.468^c^	4.51 ± 5.973^c^
C3G3	0.77 ± 0.659^c^	2.54 ± 7.429^c^	3.19 ± 8.525^c^

Note

C = percentage of weight‐volumetric solution of bitter orange peel extract, G = Gaz‐angubin (%) (C1 = 0.025, C2 = 0.05; C3 = 0.075); (G1 = 5, G2 = 10; G3 = 15).

* Means with different subscripts differ significantly (*p* < .05).

During storage, TMC increased in the flavored milk samples (*p* < .01), which can be attributed to reduced polyphenol content of them. Most phenolic compounds are in bond with other compounds such as proteins, and only a small part of phenolic compounds is in a free form (Bind et al., 2014). Therefore, it can be stated that their antibacterial and antifungal properties are limited. On the other hand, it seems that with reduction of pH and increasing cold storage time, the solubility of polyphenol compounds decreases; they then precipitate, where the inhibition properties of polyphenols against bacteria decline (Kazemizadeh & Fadaei, 2016). Also, Kazemizadeh and Fadaei (2016) reported increased SPC in flavored milks containing date syrup and pomegranate peel extract during storage time.

### 3.5 Overall acceptability of flavored milk samples during cold storage

There is a significant difference between overall acceptability of the different treatments (Table 6). In all of the samples, with increased percentages of Gaz‐angubin and bitter orange peel extract, the overall acceptability score increased. This score decreased following production in all samples on the fifth and tenth days over time, except for the sample containing 15% Gaz‐angubin and 0.075% bitter orange peel extract (C3G3), though this reduction is trivial in the sample containing 15% Gaz‐angubin and 0.025% bitter orange peel extract (C1G3). Keshtkaran et al. (2012) reported that elevation of viscosity had a positive effect on the increasing of overall acceptability score in date syrup drink. In this research, elevation of viscosity also affected the increasing of overall acceptability score. Further, Dalim et al. (2012) confirmed that the overall acceptability score in banana flavored milk, when compared with chikoo flavored milk, was greater due to its higher viscosity. Also, Hasani and Javadian (2016) reported no undesirable changes in sensorial attributes of common carp fillets treated with high concentration (1%) of the encapsulated bitter orange peel extract.

**TABLE 6:** Overall acceptability of the flavored milk samples containing bitter orange peel extract and Gaz‐angubin during cold storage (mean ± *SD*)*

Day Treatment	0	5	10
C1G1	4.4 ± 0.7^abcd^	3.9 ± 0.57^bcde^	1.6 ± 0.7^j^
C1G2	4.6 ± 0.7^ab^	3.8 ± 0.42^cde^	3.7 ± 0.82^de^
C1G3	4.8 ± 0.13^a^	4.5 ± 0.7^abc^	3.9 ± 1.1^bcde^
C2G1	2.9 ± 1.52^fghi^	2.7 ± 0.26^ghi^	2.5 ± 1.43^hi^
C2G2	3.5 ± 0.85^ef^	3.4 ± 0.52^egh^	2.7 ± 0.82^ghi^
C2G3	3.2 ± 0.79^efgh^	2.8 ± 0.79^fghi^	2.4 ± 0.52^i^
C3G1	4.5 ± 0.7^abc^	3.2 ± 1.75^efgh^	2.9 ± 0.1^fghi^
C3G2	4.7 ± 0.15^a^	3.3 ± 0.48^efg^	3.2 ± 0.63^efgh^
C3G3	4.8 ± 0.42^a^	4.7 ± 0.48^a^	4.3 ± 0.67^abcd^

Note

C = percentage of weight‐volumetric solution of bitter orange peel extract, G = Gaz‐angubin (%) (C1 = 0.025, C2 = 0.05; C3 = 0.075); (G1 = 5, G2 = 10; G3 = 15).

* Means with different subscripts differ significantly (*p* < .05).

## 4 CONCLUSION

In this study, Gaz‐angubin and bitter orange peel extract were used for the production of flavored milk, and some properties of the produced flavored milk samples during storage at 4°C were evaluated. This study showed that increasing the percentage of Gaz‐angubin and bitter orange peel extract increased viscosity, antioxidant activity, and TPC and improved the sensory characteristics. Using Gaz‐angubin in milk resulted in increased acceptability by consumers, due to its unique sweet taste, which is mainly due to fructose. Gaz‐angubin and bitter orange peel extract had a significant inhibitory effect on microbial growth. Therefore, these compounds can be a good alternative to synthetic materials to preserve food products through prevention oxidation of fat during storage. In general, the flavored milk sample containing 15% Gaz‐angubin and 0.075% bitter orange peel extract claimed the maximum percentage of inhibition of free radicals and TPC, the lowest TMC and the highest overall acceptability score.

## CONFLICT OF INTEREST

The authors declare that there is no conflict of interest.
